# Renoprotective Effect of Agalsidase Alfa: A Long-Term Follow-Up of Patients with Fabry Disease

**DOI:** 10.3390/jcm11164810

**Published:** 2022-08-17

**Authors:** Markus Cybulla, Kathleen Nicholls, Sandro Feriozzi, Aleš Linhart, Joan Torras, Bojan Vujkovac, Jaco Botha, Christina Anagnostopoulou, Michael L. West

**Affiliations:** 1 Center of Internal Medicine, Department of Nephrology and Rheumatology, Nephrologicum Markgräflerland MVZ, 79379 Müllheim, Germany; 2 Department of Nephrology, Royal Melbourne Hospital, Parkville, VIC 3050, Australia; 3 Department of Medicine, University of Melbourne, Parkville, VIC 3010, Australia; 4 Nephrology and Dialysis Unit, Belcolle Hospital, 01100 Viterbo, Italy; 5 Second Department of Medicine, Department of Cardiovascular Medicine, First Faculty of Medicine, Charles University and General University Hospital, 12808 Prague, Czech Republic; 6 Institut d’Investigació Biomèdica de Bellvitge (IDIBELL), L’Hospitalet de Llobregat, 08908 Barcelona, Spain; 7 Faculty of Medicine, Campus Bellvitge, University of Barcelona, L’Hospitalet de Llobregat, 08907 Barcelona, Spain; 8 Department of Internal Medicine, Slovenj Gradec General Hospital, 2380 Slovenj Gradec, Slovenia; 9 Takeda Pharmaceuticals International AG, 8152 Zurich, Switzerland; 10 Department of Medicine, Dalhousie University, Halifax, NS B3H 4R2, Canada

**Keywords:** Fabry disease, proteinuria, estimated glomerular filtration rate, enzyme replacement therapy

## Abstract

Fabry disease is a rare lysosomal storage disorder caused by mutations in the GLA gene, which, without treatment, can cause significant renal dysfunction. We evaluated the effects of enzyme replacement therapy with agalsidase alfa on renal decline in patients with Fabry disease using data from the Fabry Outcome Survey (FOS) registry. Male patients with Fabry disease aged >16 years at agalsidase alfa start were stratified by low (≤0.5 g/24 h) or high (>0.5 g/24 h) baseline proteinuria and by ‘classic’ or ‘non-classic’ phenotype. Overall, 193 male patients with low (*n* = 135) or high (*n* = 58) baseline proteinuria were evaluated. Compared with patients with low baseline proteinuria, those with high baseline proteinuria had a lower mean ± standard deviation baseline eGFR (89.1 ± 26.2 vs. 106.6 ± 21.8 mL/min/1.73 m^2^) and faster mean ± standard error eGFR decline (−3.62 ± 0.42 vs. −1.61 ± 0.28 mL/min/1.73 m^2^ per year; *p* < 0.0001). Patients with classic Fabry disease had similar rates of eGFR decline irrespective of baseline proteinuria; only one patient with non-classic Fabry disease had high baseline proteinuria, preventing meaningful comparisons between groups. In this analysis, baseline proteinuria significantly impacted the rate of eGFR decline in the overall population, suggesting that early treatment with good proteinuria control may be associated with renoprotective effects.

## 1. Introduction

Fabry disease is a rare X-linked inherited lysosomal storage disorder caused by mutations in the GLA gene that encodes the alpha-galactosidase A (α-Gal A) enzyme [1]. Abnormal or deficient enzyme activity leads to intracellular accumulation of glycosphingolipids—predominantly globotriaosylceramide (Gb3)—in cells throughout the body, resulting in multisystemic organ manifestations [2]. The most common clinical signs and symptoms include acroparaesthesia, angiokeratomas, hypohidrosis, gastrointestinal symptoms, corneal and lenticular opacities, and major end-organ damage with involvement of the kidneys, heart, and brain [2]. Fabry disease can be classified into two types: classic disease, which presents as a severe early-onset phenotype with multiorgan involvement, and a late-onset (or variant) phenotype, which is generally milder with major involvement of a single organ system, most commonly cardiac [3]. Enzyme replacement therapy (ERT) with agalsidase alfa or agalsidase beta is the mainstay of treatment and has been shown to slow renal and cardiac disease progression [4,5,6,7,8]. In addition to ERT, the α-Gal A pharmacological chaperone migalastat can be used for the treatment of adults with Fabry disease and an amenable GLA mutation [9].

Renal manifestations of Fabry disease have been shown to occur in most males with the disease, with proteinuria and renal impairment appearing between the second and fifth decade of life and progressing to end-stage renal disease [10]. Signs and symptoms of renal impairment in Fabry disease are preceded by morphological changes, and in one study, microscopic assessment of renal morphology revealed signs of glomerular and vascular changes in children aged 7–18 years, despite normal renal function [11]. Patients with Fabry disease and renal dysfunction are also more likely to present with cardiac symptoms—predominantly left ventricular hypertrophy accompanied by cardiac fibrosis—contributing to an overall poorer prognosis [12].

The extent of renal dysfunction prior to treatment initiation, the degree of proteinuria, and the disease phenotype independently influences the rate of renal decline in patients with Fabry disease [13,14]. Advanced renal disease with higher levels of proteinuria (urinary protein to creatinine ratio > 0.5 g/g) and lower estimated glomerular filtration rate (eGFR) have been more frequently observed in male patients with the more severe classic disease compared with male patients with the milder non-classic disease (hazard ratio (HR) (95% confidence intervals (CI)): 9.24 [1.73–49.45]) and with female patients with classic disease (HR: 9.07 [1.96–25.13]) [3]. Furthermore, high renal involvement (defined using the same criteria) was associated with a rapid decline in renal function in a study of 52 patients with Fabry disease [13]. In this study, patients with high renal involvement at the time of ERT initiation had a mean annual rate of change in eGFR of −6.82 mL/min/1.73 m^2^ compared with −1.89 mL/min/1.73 m^2^ in patients with low renal involvement (urine protein/creatinine ratio ≤ 0.5 g/g and <50% sclerotic glomeruli) at baseline, suggesting that initiation of ERT prior to the occurrence of irreversible organ damage may result in more favorable outcomes [13].

The Fabry Outcome Survey (FOS) registry (Clinicaltrials.gov NCT03289065, accessed on 7 August 2022; sponsored by Shire, a Takeda company, Lexington, MA, USA) is an international multicenter disease registry with over 20 years of data from treated and untreated patients with a confirmed diagnosis of Fabry disease.

Given that few studies have evaluated the effect of disease phenotype (classic vs. non-classic) and the extent of baseline renal dysfunction on the long-term rate of progression of renal impairment, we evaluated the impact of urinary proteinuria on eGFR in patients with classic versus non-classic Fabry disease treated with agalsidase alfa using data from FOS.

## 2. Materials and Methods

This analysis evaluated data collected from FOS for male patients who met the following inclusion criteria: treated only with agalsidase alfa for ≥5 years; no other ERT prior to FOS entry; >16 years of age at the start of agalsidase alfa treatment; no history of dialysis/transplantation prior to agalsidase alfa initiation, an eGFR of ≥45 mL/min/1.73 m^2^ at baseline, calculated using the Chronic Kidney Disease Epidemiology Collaboration (CKD-EPI) equation; urinary protein measured at baseline. FOS data used for these analyses were extracted for the period from database inception in 2001 to 7 January 2022. Patients aged <16 years at treatment start were excluded from this analysis owing to the CDK-EPI formula for calculation of eGFR being inappropriate in this population.

Patients are enrolled in FOS on a voluntary basis and are managed under the direction of their physician in accordance with routine clinical practice. FOS was approved by the ethics institutional review boards of the participating centers. Further, this registry was compliant with relevant global and local regulations and best practices: Good Pharmacoepidemiological Practice and Good Research for Comparative Effectiveness principles. The relevant principles of the International Council for Harmonisation Good Clinical Practice (ICH GCP) guidelines (ICH E6) were followed as appropriate for an observational registry, consistent with the Declaration of Helsinki. All participants gave written informed consent.

Data on patient demographics, clinical characteristics, and renal and selected cardiac endpoints were collected via the FOS web-based electronic case report form for the period from database inception in 2001 to 7 January 2022. Baseline was defined as the value with the date closest to treatment initiation within a window of −6 to +3 months. Endpoints included eGFR calculated using the CKD-EPI equation [15], urinary protein levels, and arterial blood pressure control.

Patients were stratified by level of urinary protein at baseline, with low proteinuria defined as ≤0.5 g/24 h and high proteinuria defined as >0.5 g/24 h. Patients with available data were further stratified by GLA variants into those associated with classic phenotypes and those associated with non-classic phenotypes (inclusive of N215S, predominantly known as a cardiac variant [16] and IVS4+919G>A, but exclusive of D313Y [17], variants of undetermined significance, those likely benign or non-pathogenic, and those for which no definite phenotype could be assigned based on the literature). Variants were assigned to a phenotype based on multiple published sources [18,19,20,21,22,23,24,25,26,27,28,29,30,31], the fabry-database.org GLA variant database [32], and the International Fabry Disease Genotype-Phenotype Database (dbFGP) [33]. GLA genotype was determined by molecular analysis and input into the FOS database by healthcare personnel at participating centers. A further subgroup analysis was undertaken of patients who progressed to dialysis or transplantation within the follow-up period.

Patient characteristics at baseline were summarized using descriptive statistics. Between-group comparisons of baseline data were made using the chi-squared test for categorical variables and two-sample t-test for continuous variables. Mean annual rates of change in eGFR and urinary protein were calculated using mixed-effects linear regression analysis for patients with at least three measurements recorded in FOS. Values were restricted to 5–150 mL/min/1.73 m^2^ for CKD-EPI and 0–5 g/24 h for urinary protein. Values outside these ranges were considered as missing. Statistical analyses were performed using SAS statistical software V.9.4 (SAS Institute, Cary, NC, USA).

## 3. Results

### 3.1. Patient Characteristics at Baseline

Of a total of 1857 participants enrolled in FOS as of 7 January 2022, 918 were male and 193 met the inclusion criteria and were included in the analyses; 135 patients had baseline proteinuria ≤ 0.5 g/24 h and 58 patients had baseline proteinuria > 0.5 g/24 h. Overall mean age at diagnosis was 28.9 years and age at treatment initiation was 36.6 years (*n* = 193) (Table 1). Patients received treatment with agalsidase alfa for a mean of 13.0 years and 11.9 years in the low and high baseline proteinuria groups, respectively. There were no significant differences in baseline systolic and diastolic blood pressure between the low and the high proteinuria groups, and the proportion of patients with controlled blood pressure at baseline (<120/80 mm Hg) was similar in both groups (26.5% and 24.1%, respectively; Table 1). Mean baseline eGFR was higher for patients with low versus high baseline proteinuria (106.6 vs. 89.1 mL/min/1.73 m^2^, respectively, *p* < 0.001; Table 1).

### 3.2. Baseline Patient Characteristics by Variant Classification

Sufficient genetic data for variant classification were available for 103 of 193 patients (53.4%). Of these, 71 (68.9%) had variants associated with the classic phenotype (47 low and 24 high baseline proteinuria) and 20 (19.4%) had variants associated with a non-classic phenotype (19 low and 1 high baseline proteinuria). A further 12 patients had variants associated with no definite phenotype or were likely non-pathogenic and were excluded from analyses by genotype. Individual variants and phenotype classifications are listed in Supplemental Table S1.

Overall, patients with the classic phenotype were younger at diagnosis (mean ± SD: 22.2 ± 10.5 years; *n* = 71) and at treatment initiation (32.6 ± 9.3 years; *n* = 71) than patients with a non-classic phenotype (48.2 ± 15.0 years (*n* = 20), and 50.2 ± 14.2 years (*n* = 20), respectively). Additionally, patients with the classic phenotype had a higher eGFR and higher urinary protein than patients with a non-classic phenotype (106.7 ± 22.0 vs. 91.5 ± 19.0 mL/min/1.73 m^2^ and 0.50 vs. 0.17 g/24 h, respectively). Within the classic phenotype subgroup, patients with high baseline proteinuria were older at diagnosis and treatment start, with higher urinary protein and lower eGFR at baseline than those with low baseline proteinuria (Table 2).

### 3.3. Baseline Patient Characteristics by Progression to ESRD

Eighteen patients received dialysis or renal transplantation during the analysis period. Of these, 13 (72.2%) had high proteinuria at baseline compared with 30.1% (58/193) of the overall population. Nine of 18 patients (50%) had available genetic data, all of whom had variants associated with the classic genotype (3 with low baseline proteinuria and 6 with high baseline proteinuria), compared with 68.9% (71/103) of the overall population. Mean age at treatment initiation was 39.8 years for those with low baseline proteinuria and 39.2 years for those with high baseline proteinuria, and the mean time from treatment start to dialysis or transplant was 14.4 and 8.3 years, respectively. Age at treatment initiation was similar for both those receiving dialysis/renal transplantation and those who did not, suggestive of rapid deterioration of renal function in the high proteinuria subgroup (Table 3). At baseline, patients receiving dialysis/transplantation had lower eGFR and higher urinary protein than their respective non-dialysis/transplantation subgroups (Table 3).

### 3.4. Change in Estimated Glomerular Filtration Rate

**Figure 1:**
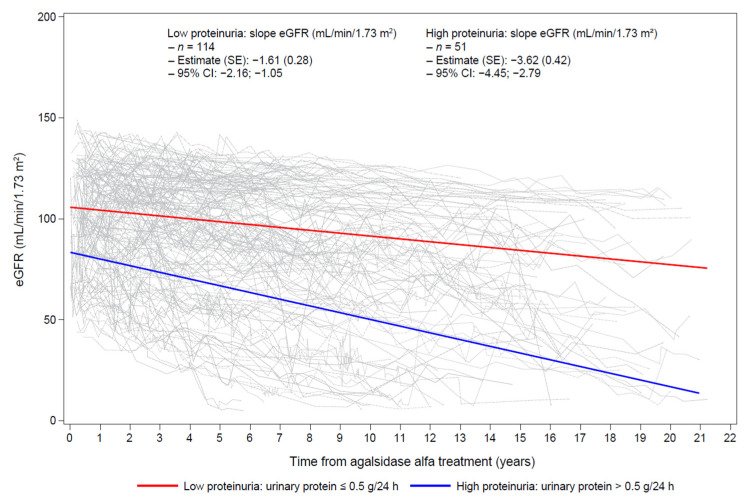
Change in eGFR over time by level of proteinuria at baseline. eGFR was measured using the CKD-EPI equation. CI—confidence interval; CKD-EPI—Chronic Kidney Disease Epidemiology Collaboration; eGFR—estimated glomerular filtration rate; SE, standard error.

A total of 165 patients had at least three eGFR measurements over the follow-up period. Mean annual rates of decline in eGFR were −1.61 mL/min/1.73 m^2^ for patients with low baseline proteinuria (*n* = 114) and −3.62 mL/min/1.73 m^2^ for patients with high baseline proteinuria (*n* = 51; *p* < 0.0001; Table 4), indicating a faster rate of worsening of renal impairment in the latter group (Figure 1).

**Figure 2:**
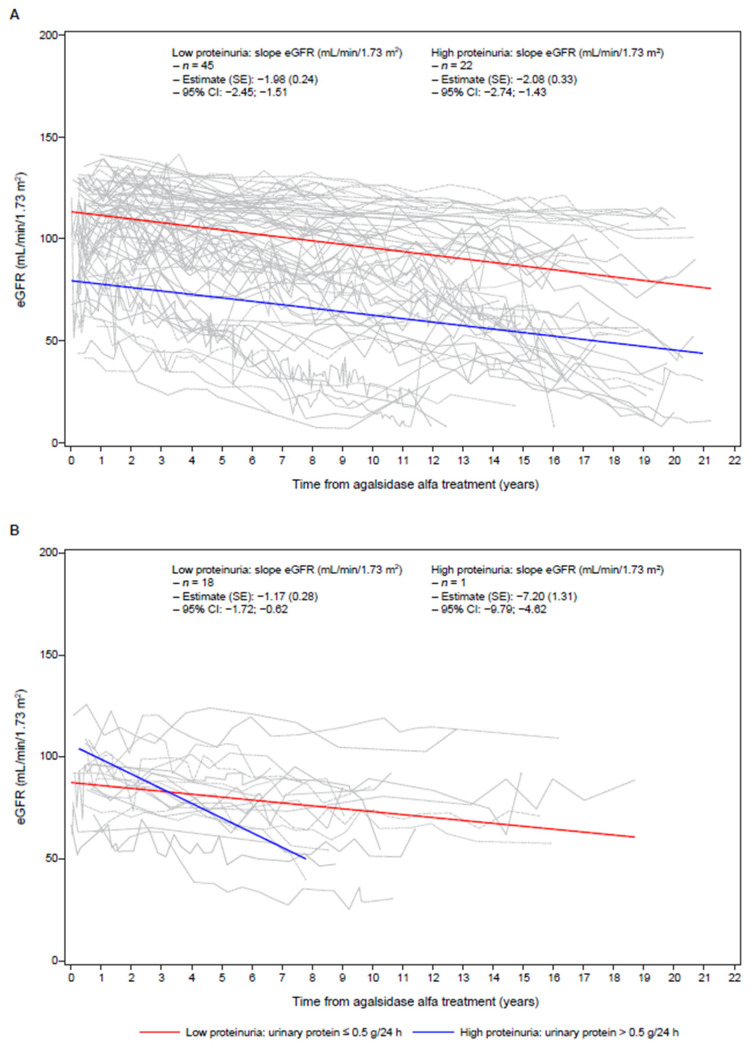
Change in eGFR over time by mutation subgroup and level of proteinuria at baseline. (**A**) Classic mutations. (**B**) Non-classic mutations. eGFR was measured using the CKD-EPI equation. CI—confidence internal; CKD-EPI—Chronic Kidney Disease Epidemiology Collaboration; eGFR—estimated glomerular filtration rate; SE—standard error.

In total, 86 patients with available genetic information had at least three eGFR measurements: 63 patients with low baseline proteinuria (*n* = 45 classic; *n* = 18 non-classic) and 23 patients with high baseline proteinuria (*n* = 22 classic; *n* = 1 non-classic). Patients with low proteinuria and a non-classic phenotype had the slowest mean eGFR decline of −1.17 mL/min/1.73 m^2^ (*n* = 18; Figure 2B), whereas patients with the classic phenotype had similar rates of eGFR decline irrespective of baseline proteinuria (−1.98 and −2.08 mL/min/1.73 m^2^ for the low proteinuria (*n* = 45) and high proteinuria (*n* = 22) groups, respectively, *p* = 0.8069; Figure 2A). One patient with a non-classic phenotype and high baseline proteinuria of 0.71 g/24 h had a rate of eGFR decline of −7.20 mL/min/1.73 m^2^ (Table 4; Figure 2B); however, this patient had minimal impact on the overall rate of decline in the high baseline proteinuria subgroup (−3.55 mL/min/1.73 m^2^ excluding this patient (*n* = 50) compared with −3.62 mL/min/1.73 m^2^ inclusive (*n* = 51)). Variants and phenotypes associated with rapid (≤−3 mL/min/1.73 m^2^) or slow (>−3 mL/min/1.73 m^2^) rates of eGFR decline are listed in Supplemental Table S1.

Greater average rates of eGFR decline were observed for patients undergoing dialysis or transplantation compared with the overall population, although statistical significance was achieved with the high baseline proteinuria group only owing to small patient numbers and high variability. For dialysis/transplant patients, mean ± SD rates of decline were −3.56 ± 2.99 mL/min/1.73 m^2^ (*n* = 5) and −6.28 ± 1.96 mL/min/1.73 m^2^ (*n* = 12) for low and high baseline proteinuria groups, respectively, compared with −1.61 ± 0.28 and −3.62 ± 0.42 mL/min/1.73 m^2^ in the low and high baseline proteinuria overall groups.

### 3.5. Changes in Urinary Protein

A total of 121 patients had at least three urinary protein measurements over the follow-up period: 82 with low baseline proteinuria (including three who underwent dialysis or transplantation) and 39 with high baseline proteinuria (including 10 who underwent dialysis or transplantation). The mean annual rate of increase in urinary protein levels was 0.02 g/24 h in the low proteinuria group (*n* = 82; *p* = 0.0028) and 0.03 g/24 h in the high baseline proteinuria group (*n* = 39; *p* = 0.0222), with no significant difference between the low and high proteinuria groups (*p* = 0.7531). Change in urinary protein was similar for patients with classic and non-classic mutations in both the low and high baseline proteinuria groups (Table 4) and for patients who underwent dialysis or transplantation.

## 4. Discussion

In this evaluation of data from FOS, patients with high urinary proteinuria at baseline (time of treatment initiation) had significantly lower baseline eGFR and more rapid eGFR decline over time compared with patients with low urinary proteinuria at baseline, suggestive of renal impairment prior to treatment initiation for this group of patients. In our study, baseline proteinuria levels had no impact on the rate of eGFR decline in patients with the classic phenotype, although patients in the classic low proteinuria subgroup had markedly higher baseline eGFR and were markedly younger at treatment initiation than those with high proteinuria, suggesting that patients with low proteinuria may have been detected earlier, while renal function remained relatively conserved, perhaps as a result of non-renal manifestations of Fabry disease. The effect of baseline proteinuria on patients with non-classic phenotype was undetermined owing to small patient numbers in the high proteinuria subgroup. The scarcity of patients in this subgroup may reflect a low risk of proteinuria associated with the non-classic phenotype, which may be more typically associated with cardiac or cerebrovascular manifestations. One patient in this subgroup in our analysis started treatment within 3 years of diagnosis and had both high proteinuria and low eGFR at baseline, indicating the possibility of existing non-Fabry disease related renal dysfunction.

Within the low proteinuria group, the classic phenotype was associated with more rapid eGFR decline than non-classic phenotypes, in line with previous studies, which reported rates of eGFR decline of −2.7 to −2.93 mL/min/1.73 m^2^ among males, most or all who had classic Fabry disease [6,14,34] and −1.54 to −1.8 mL/min/1.73 m^2^ for males with non-classic Fabry disease [6,34]. In the current analysis, patients with a classic phenotype were younger at diagnosis and treatment initiation and had a higher mean urinary protein concentration than patients with a non-classic phenotype, indicative of a more severe disease state at a younger age, consistent with previous studies [3,6,34].

These findings are largely consistent with previous reports. A post-marketing surveillance study of agalsidase alfa-treated patients with Fabry disease in Japan (Sasa et al., 2019 [6]) reported rates of eGFR decline of −0.95 to −0.98 mL/min/1.73 m^2^ in 238 male patients with baseline proteinuria of <0.3 g/24 h, and −3.24 to −4.52 mL/min/1.73 m^2^ in 56 male patients with baseline proteinuria of >0.3 g/ 24 h. This study included a similar proportion of patients with classic mutations (approximately 80%); however, although age at diagnosis was similar between the two studies, treatment was initiated more rapidly after diagnosis in the Sasa et al., study than in our analysis. Further, the proteinuria cutoff was lower than used in the current analysis, likely contributing to the slower rate of eGFR decline in patients with low proteinuria.

A study of 52 Danish patients with Fabry disease treated with ERT (Madsen et al., 2019 [35]) reported a rate of decline in eGFR of −0.8 mL/min/1.73 m^2^ per year in a cohort of patients with baseline urinary protein of 0.2 g/24 h and an eGFR of 97.0 mL/min/1.73 m^2^. Although baseline proteinuria and eGFR were similar to those of the low proteinuria group in our study, we observed a considerably faster decline in eGFR of −1.61 mL/min/1.73 m^2^. The difference between the rates of eGFR decline may be explained, at least in part, by the exclusion of patients with end-stage renal disease and the shorter duration of follow-up in the Madsen et al., study compared with our analysis (median 7 vs. 12.3 years (low baseline proteinuria group)) [35].

Lastly, a study by Germain et al., (2015) [13] of patients treated with agalsidase beta observed an annual rate of change in eGFR of −1.89 mL/min/1.73 m^2^ for patients with low renal involvement at baseline, which is closer to values observed for the low proteinuria cohort in the current analysis [13]. In the Germain et al., study, low renal involvement was defined as both a urine protein/creatinine ratio ≤ 0.5 g/g and <50% sclerotic glomeruli assessed from clinical study biopsies, while the current study stratified patients by urinary proteinuria at baseline ≤ 0.5 g/24 h, necessitating caution in comparisons between the two cohorts. The Germain et al., study also reported an annual decline in eGFR of −6.82 mL/min/1.73 m^2^ for patients with high renal involvement, defined as a urine protein/creatinine ratio > 0.5 g/g and ≥50% sclerotic glomeruli [13], compared with the smaller decline of −3.62 mL/min/1.73 m^2^ in patients with high proteinuria in the current analysis. However, all patients included in the Germain et al., study had classic mutations, in contrast to 78% of patients in the current study, which may have contributed to the difference in rates. Further, although mean baseline urinary protein levels and age at treatment initiation were similar for the high proteinuria/high renal involvement populations of both studies, the mean duration of follow-up was shorter in the Germain et al., study (8.4 vs. 11.9 years (high baseline proteinuria group) in the current analyses) and mean ± SD eGFR at baseline was higher in the earlier study (101.6 ± 22.78 vs. 89.1 ± 26.2 mL/min/1.73 m^2^ per year (high baseline proteinuria group) in the current analysis) [13].

While eGFR has historically been viewed as the best overall marker for renal disease [36], eGFR rates can worsen rapidly toward the end stages of renal disease, providing an imbalanced assessment of change over time, while additional factors may influence the overall risk of renal decline [10]. This was illustrated by a subgroup of 18 patients who progressed to dialysis or renal transplantation after starting ERT. Within this group, overall mean (SD) eGFR at baseline was 84.2 (29.8) mL/min/1.73 m^2^ compared with 103.1 (24.8) mL/min/1.73 m^2^ for those who did not receive dialysis or transplantation, suggestive of renal impairment prior to treatment initiation in this group. These dialysis/transplant patients may represent a group of ‘fast renal progressors’ with rapid deterioration in renal function after treatment initiation.

A few limitations should be considered when evaluating the data from this study. Female patients with Fabry disease were excluded from this analysis, with data presented for the more highly affected male population only. Although impaired renal function is well described in female heterozygotes, progression to end-stage renal failure is infrequent, with only 1–2% of female patients requiring dialysis of transplantation [2,37]. Low patient numbers in the high proteinuria and non-classic phenotype groups mean these data should be interpreted with caution. This analysis did not investigate other factors that influence the progression of renal dysfunction and eGFR slope following ERT, such as the presence of cardiovascular risk factors, the use of renin–angiotensin system (RAS) inhibitors, vitamin D supplements, or of dietary sodium intake, because a large proportion of the concomitant medication data for patients in FOS are missing a finite start date. Although, due to ERT having no effect on proteinuria, we could speculate that good proteinuria control was achieved in our cohort by antiproteinuric therapy, most likely RAS inhibitors. Further limitations relate to the nature of retrospective analyses of registry data, and data completeness may vary across participating centers. For example, histological data were not collected in the FOS registry; therefore, histological verification of renal impairment was not possible. Finally, there is the possibility of patient enrollment bias, as patients with more severe symptoms or who are receiving treatment may be more likely to enroll in a registry. Despite this, the studied population is sufficiently large to allow us to glimpse the features that occur in Fabry nephropathy.

## 5. Conclusions

In this study, the initiation of ERT in patients with low baseline proteinuria was associated with slower renal decline in comparison with patients initiating ERT with high baseline proteinuria. These findings suggest that the presence of proteinuria may reflect renal/glomerular damage over time and may act as a prognostic factor for renal decline in Fabry disease. Early/prompt treatment with ERT as well as with adjuvant therapy for control of proteinuria —before the occurrence of significant morphological changes and elevated proteinuria—may be associated with renoprotective effects, resulting in the stabilization of renal function relative to the expected decline in patients with Fabry disease. Active monitoring and early treatment are warranted for optimal benefit, particularly for patients with early onset of symptoms, to help prevent or delay the renal progression found in patients with Fabry disease.

## Figures and Tables

**Table 1:** Patient characteristics by level of proteinuria at baseline.

Characteristic	Low Proteinuria (Baseline Urinary Protein ≤ 0.5 g/24 h) *n* = 135	High Proteinuria (Baseline Urinary Protein > 0.5 g/24 h) *n* = 58	Total *n* = 193
Age at diagnosis, years			
Mean ± SD	28.5 ± 16.2	29.6 ± 13.7	28.9 ± 15.5
Median (range)	24.0 (2.0–75.0)	27.0 (8.0–71.0)	26.0 (2.0–75.0)
Age at treatment initiation, years			
Mean ± SD	35.3 ± 13.9	39.7 ± 10.4 ^‡^	36.6 ± 13.1
Median (range)	32.1 (16.8–75.9)	39.9 (17.3–71.5)	35.3 (16.8–75.9)
Duration of treatment, years			
Mean ± SD	13.0 ± 4.9	11.9 ± 5.2	12.6 ± 5.0
Median (range)	12.3 (5.0–21.6)	10.6 (5.1–20.9)	11.9 (5.0–21.6)
Blood pressure, mm Hg			
*n* (missing)	117 (18)	54 (4)	171 (22)
Mean ± SD systolic BP	125.9 ± 15.8	126.3 ± 16.8	126.1 ± 16.1
Mean ± SD diastolic BP	74.5 ± 11.1	76.0 ± 11.4	75.0 ± 11.2
Blood pressure control (<120/80 mm Hg)			
*n* (missing)	117 (18)	54 (4)	171 (22)
At baseline, *n* (%)	31 (26.5)	13 (24.1)	44 (25.7)
At last available assessment, *n* (%)	45 (38.5)	25 (47.2) *	70 (41.2) ^†^
Mean ± SD time between baseline and last BP assessment, years	11.6 ± 5.5	10.7 ± 5.0 *	11.3 ± 5.4 ^†^
Urinary protein, g/24 h			
Mean ± SD	0.18 ± 0.11	1.23 ± 0.71 ^§^	0.50 ± 0.62
Median (range)	0.16 (0.0–0.5)	0.97 (0.5–3.2)	0.23 (0.0–3.23)
eGFR, mL/min/1.73 m^2^			
Mean ± SD	106.6 ± 21.8	89.1 ± 26.2 ^§^	101.3 ± 24.5
Median (range)	111.0 (54.5–144.7)	86.4 (46.6–135.4)	105.2 (46.6–144.7)
CKD stage, *n* (%)			
Hyperfiltration: ≥130 mL/min	15 (11.1)	2 (3.4)	17 (8.8)
Stage 1: eGFR 9–<130 mL/min	91 (67.4)	25 (43.1)	116 (60.1)
Stage 2: eGFR 60–<90 mL/min	27 (20.0)	21 (36.2)	48 (24.9)
Stage 3: eGFR 30–<60 mL/min	2 (1.5)	10 (17.2)	12 (6.2)
Stage 4: eGFR 15–<30 mL/min	0	0	0
Stage 5: eGFR <15 mL/min	0	0	0
Phenotype, *n* (%)	76	27	103
Classic	47 (61.8)	24 (88.9)	71 (68.9)
Non-classic	19 (25.0)	1 (3.7)	20 (19.4)
Other ^¶^	10 (13.2)	2 (7.4)	12 (11.7)

* *n* = 53. ^†^ *n* = 170. ^‡^ *p* ≤ 0.05; ^§^ *p* ≤ 0.001. ^¶^ no definite phenotype or likely non-pathogenic. BP—blood pressure; CKD—chronic kidney disease; eGFR—estimated glomerular filtration rate; SD—standard deviation.

**Table 2:** Patient characteristics by mutation type and level of proteinuria at baseline.

	Classic Mutations *n* = 71		Non-Classic Mutations *n* = 20	
	Low Proteinuria (Baseline Urinary Protein ≤ 0.5 g/24 h) *n* = 47	High Proteinuria (Baseline Urinary Protein > 0.5 g/24 h) *n* = 24	Low Proteinuria (Baseline Urinary Protein ≤ 0.5 g/24 h) *n* = 19	High Proteinuria (Baseline Urinary Protein > 0.5 g/24 h) *n* = 1
Age at diagnosis, years				
Mean ± SD	20.5 ± 9.3	25.4 ± 12.0	49.4 ± 14.4	25.0
Median (range)	21.0 (2.0–41.0)	24.5 (8.0–51.0)	54.0 (14.0–66.0)	25.0 (25.0–25.0)
Age at treatment initiation, years				
Mean ± SD	29.4 ± 8.4	38.9 ± 7.7 *	51.5 ± 13.5	27.3
Median (range)	28.1 (17.0–51.7)	37.9 (25.3–53.0)	55.8 (20.4–66.8)	27.3 (27.3–27.3)
Duration of treatment, years				
Mean ± SD	16.2 ± 4.5	15.6 ± 4.5	11.8 ± 4.1	7.8
Median (range)	17.0 (5.0–21.6)	16.7 (6.4–20.9)	10.6 (5.7–19.2)	7.8 (7.8–7.8)
Urinary protein, g/24 h				
Mean ± SD	0.20 ± 0.11	1.09 ± 0.59 *	0.14 ± 0.12	0.71 *
Median (range)	0.18 (0.06–0.48)	0.92 (0.51–2.37)	0.10 (0.02–0.50)	0.71 (0.71–0.71)
eGFR, mL/min/1.73 m^2^				
Mean ± SD	115.1 ± 16.3	90.3 ± 22.8 *	91.2 ± 19.5	97.8
Median (range)	118.5 (60.9–139.6)	89.7 (46.9–129.8)	94.8 (64.8–129.6)	97.8 (97.8–97.8)
CKD stage, *n* (%)				
Hyperfiltration: ≥130 mL/min	5 (10.6)	0	0	0
Stage 1: eGFR 9–<130 mL/min	39 (83.0)	12 (50.0)	10 (52.6)	1 (100)
Stage 2: eGFR 60–<90 mL/min	3 (6.4)	10 (41.7)	9 (47.4)	0
Stage 3: eGFR 30–<60 mL/min	0	2 (8.3)	0	0
Stage 4: eGFR 15–<30 mL/min	0	0	0	0
Stage 5: eGFR <15 mL/min	0	0	0	0

* *p* ≤ 0.001. CKD—chronic kidney disease; eGFR—estimated glomerular filtration rate; ND—not determined; SD—standard deviation.

**Table 3:** Patient characteristics by level of proteinuria at baseline and extent of renal progression to dialysis or transplant after ERT start.

Characteristic	Dialysis/Transplantation *n* = 18		No Dialysis/Transplantation *n* = 175	
	Low Proteinuria (Baseline Urinary Protein ≤ 0.5 g/24 h) *n* = 5	High Proteinuria (Baseline Urinary Protein > 0.5 g/24 h) *n* = 13	Low Proteinuria (Baseline Urinary Protein ≤ 0.5 g/24 h) *n* = 130	High Proteinuria (Baseline Urinary Protein > 0.5 g/24 h) *n* = 45
Age at diagnosis, years				
Mean ± SD	25.0 ± 19.4	31.0 ± 15.8	28.7 ± 16.2	29.2 ± 13.2
Median (range)	26.0 (5.0–55.0)	27.0 (8.0–62.0)	24.0 (2.0–75.0)	27.0 (9.0–71.0)
Age at treatment initiation, years				
Mean ± SD	39.8 ± 11.4	39.2 ± 11.6	35.1 ± 14.0	39.8 ± 10.1
Median (range)	33.2 (29.9–55.5)	38.4 (17.3–63.0)	32.0 (16.8–75.9)	39.8 (19.4–71.5)
Duration of treatment, years				
Mean ± SD	17.9 ± 3.7	12.0 ± 5.7	12.8 ± 4.8	11.8 ± 5.1
Median (range)	18.8 (12.2–21.4)	9.9 (6.2–20.6)	12.2 (5.0–21.6)	10.9 (5.1–20.9)
Time from treatment start to dialysis/transplant, years				
Mean ± SD	14.4 ± 8.6	8.3 ± 3.5	–	–
Median (range)	18.4 (0.1–20.7)	9.3 (2.0–13.4)	–	–
Urinary protein, g/24 h				
Mean ± SD	0.30 ± 0.12	1.59 ± 0.62	0.18 ± 0.11	1.12 ± 0.71
Median (range)	0.26 (0.19–0.48)	1.60 (0.53–2.53)	0.16 (0.00–0.50)	0.84 (0.51–3.23)
eGFR, mL/min/1.73 m^2^				
Mean ± SD	93.2 ± 30.0	80.8 ± 30.2	107.1 ± 21.4	91.5 ± 24.8
Median (range)	111.7 (54.5–119.9)	65.0 (46.9–131.0)	110.4 (58.3–144.7)	87.4 (46.6–135.4)

eGFR—estimated glomerular filtration rate; ERT—enzyme replacement therapy; SD—standard deviation.

**Table 4:** Mixed effect linear regression analysis of clinical endpoints by level of proteinuria at baseline.

Variable	Low Proteinuria (Baseline Urinary Protein ≤ 0.5 g/24 h)				High Proteinuria (Baseline Urinary Protein > 0.5 g/24 h)				*p*-Value ^†^
	*n*	Intercept ± SE	Slope ± SE	*p*-Value *	*n*	Intercept ± SE	Slope ± SE	*p*-Value *	
Overall									
eGFR, mL/min/1.73 m^2^	114	9.67 ± 4.00	–1.61 ± 0.28	<0.0001	51	4.15 ± 3.69	–3.62 ± 0.42	<0.0001	<0.0001
Urinary protein, g/24 h	82	0.11 ± 0.05	0.02 ± 0.01	0.0028	39	0.31 ± 0.14	0.03 ± 0.01	0.0222	0.7531
Classic mutations									
eGFR, mL/min/1.73 m^2^	45	13.05 ± 9.93	–1.98 ± 0.24	<0.0001	22	1.67 ± 8.14	–2.08 ± 0.33	<0.0001	0.8069
Urinary protein, g/24 h	36	0.13 ± 0.09	0.02 ± 0.01	0.0199	19	0.51 ± 0.20	0.02 ± 0.01	0.0939	0.9750
Non-classic mutations									
eGFR, mL/min/1.73 m^2^	18	17.60 ± 7.13	–1.17 ± 0.28	<0.0001	1	30.03 ± 10.25	–7.20 ± 1.31	<0.0001	<0.0001
Urinary protein, g/24 h	10	0.14 ± 0.06	0.01 ±0.01	0.1295	0	–	–	–	–
Dialysis/transplantation									
eGFR, mL/min/1.73 m^2^	5	22.42 ± 17.74	−3.56 ± 2.99	0.2337	12	11.21 ± 14.16	−6.28 ± 1.96	0.0014	0.4468
Urinary protein, g/24 h	3	0.25 ± 0.67	0.09 ± 0.06	0.1353	10	1.70 ± 0.98	−0.06 ± 0.04	0.1329	0.0382

* *p*-Value (calculated using the Wald test) evaluating significance in the annual rate of change over time within the low or high proteinuria groups. ^†^ *p*-Value (calculated using the Wald test) comparing the annual rate of change between low and high proteinuria groups. eGFR—estimated glomerular filtration rate; SE—standard error.

## Data Availability

The datasets, including the redacted study protocol, redacted statistical analysis plan, and individual participants data supporting the results of the study, will be made available after the publication of study results within three months from initial request to researchers who provide a methodologically sound proposal. The data will be provided after de-identification, in compliance with applicable privacy laws, data protection, and requirements for consent and anonymization.

## Supplementary Materials

The following supporting information can be downloaded at: https://www.mdpi.com/article/10.3390/jcm11164810/s1, Table S1. Listing of individual mutations and classifications for patients with genetic ICF.
