# Supplementary material for: Renoprotective Effect of Agalsidase Alfa: A Long-Term Follow-Up of Patients with Fabry Disease
Source: J Clin Med. 2022 Aug 17;11(16):4810. doi: 10.3390/jcm11164810 (PMC9410255; doi:10.3390/jcm11164810)
Supplement: Supplementary file 1 [file jcm-11-04810-s001.zip › jcm-1818880-supplementary.pdf]

Supplemental Table S1. Listing of individual mutations and classifications for patients with genetic ICF.

**Patient**	**Age category at ERT Start, years**	**Phenotype**	**Mutation**	**eGFR Decline, mL/min/1.73 m^2 per year**
**Low baseline proteinuria**				
1	≥20 to <30	Classic	M284T	≤–3
2	≥20 to <30	Classic	deletion	≤–3
3	≥30 to <40	Classic	W81X	≤–3
4	≥30 to <40	Classic	A143P	≤–3
5	≥30 to <40	Classic	C56Y	≤–3
6	≥40 to <50	Classic	R342Q	≤–3
7	<20	Classic	deletion	≤–3
8	≥20 to <30	Classic	R301G	≤–3
9	≥30 to <40	Classic	L131P	≤–3
10	≥20 to <30	Classic	deletion	≤–3
11	≥30 to <40	Classic	W209X	≤–3
12	≥20 to <30	Classic	deletion	>–3
13	≥20 to <30	Classic	deletion	>–3
14	≥20 to <30	Classic	C172Y	>–3
15	≥20 to <30	Classic	C172Y	>–3
16	≥20 to <30	Classic	R227X	>–3
17	≥30 to <40	Classic	insertion	>–3
18	≥20 to <30	Classic	deletion	>–3
19	≥40 to <50	Classic	L89P	>–3
20	≥50 to <60	Classic	A143P	>–3
21	≥20 to <30	Classic	A143P	>–3
22	<20	Classic	G325S	>–3
23	<20	Classic	insertion	>–3
24	<20	Classic	deletion	>–3
25	≥20 to <30	Classic	deletion	>–3
26	<20	Classic	deletion	>–3
27	≥40 to <50	Classic	deletion	>–3
28	≥40 to <50	Classic	R220X	>–3
29	≥20 to <30	Classic	deletion	>–3
30	≥20 to <30	Classic	W24X	>–3
31	≥50 to <60	Classic	R342Q	>–3
32	≥20 to <30	Classic	R301X	>–3
33	≥20 to <30	Classic	G361R	>–3
34	≥30 to <40	Classic	insertion	>–3
35	≥20 to <30	Classic	C56Y	>–3
36	≥40 to <50	Classic	R301X	>–3
37	≥30 to <40	Classic	R220X	>–3
38	≥20 to <30	Classic	insertion	>–3
39	≥20 to <30	Classic	R227Q	>–3
40	≥20 to <30	Classic	Q157X	>–3
41	≥30 to <40	Classic	deletion	>–3
42	≥30 to <40	Classic	M187R	>–3

43	≥20 to <30	Classic	Q357X	>–3
44	<20	Classic	R342X	>–3
45	≥30 to <40	Classic	M42V	>–3
46	≥30 to <40	Classic	V316E	>–3
47	≥20 to <30	Classic	N224D	>–3
48	≥60	Non-Classic	N215S	≤–3
49	≥60	Non-Classic	N215S	≤–3
50	≥50 to <60	Non-Classic	F113L	≤–3
51	≥60	Non-Classic	IVS4+919G>A	≤–3
52	≥50 to <60	Non-Classic	IVS4+919G>A	≤–3
53	≥30 to <40	Non-Classic	N215S	>–3
54	≥50 to <60	Non-Classic	N215S	>–3
55	≥60	Non-Classic	N215S	>–3
56	≥50 to <60	Non-Classic	N215S	>–3
57	≥20 to <30	Non-Classic	L300P	>–3
58	≥50 to <60	Non-Classic	N215S	>–3
59	≥50 to <60	Non-Classic	N215S	>–3
60	≥30 to <40	Non-Classic	P205S	>–3
61	≥50 to <60	Non-Classic	IVS4+919G>A	>–3
62	≥60	Non-Classic	IVS4+919G>A	>–3
63	≥60	Non-Classic	IVS4+919G>A	>–3
64	≥50 to <60	Non-Classic	IVS4+919G>A	>–3
65	≥20 to <30	Non-Classic	deletion	>–3
66	≥40 to <50	Non-Classic	N215S	>–3
67	≥40 to <50	GVUS/Likely Benign	Q333X	>–3
68	≥60	GVUS/Likely Benign	A143T	>–3
69	≥20 to <30	No definite	C63Y	>–3
70	≥30 to <40	No definite	I232T	>–3
71	≥50 to <60	No definite	P265L	>–3
72	≥30 to <40	No definite	L36F	>–3
73	≥20 to <30	No definite	R363P	>–3
74	<20	No definite	L372P	>–3
75	≥50 to <60	No definite	R363C	>–3
76	≥20 to <30	No definite	T194I	>–3
**High baseline proteinuria**				
77	≥30 to <40	Classic	deletion	≤–3
78	≥30 to <40	Classic	A143P	≤–3
79	≥40 to <50	Classic	S345P	≤–3
80	≥40 to <50	Classic	A143P	≤–3
81	≥30 to <40	Classic	deletion	≤–3
82	≥30 to <40	Classic	IVS3+1G>A	≤–3
83	≥30 to <40	Classic	deletion	≤–3
84	≥30 to <40	Classic	deletion	≤–3
85	≥20 to <30	Classic	deletion	≤–3
86	≥50 to <60	Classic	N320I	≤–3
87	≥30 to <40	Classic	I407K	>–3
88	≥40 to <50	Classic	R227X	>–3

89	≥30 to <40	Classic	R301P	>–3
90	≥50 to <60	Classic	Y216C	>–3
91	≥40 to <50	Classic	deletion	>–3
92	≥40 to <50	Classic	W81X	>–3
93	≥20 to <30	Classic	Y134S	>–3
94	≥40 to <50	Classic	A143P	>–3
95	≥50 to <60	Classic	A143P	>–3
96	≥20 to <30	Classic	W349X	>–3
97	≥30 to <40	Classic	R227X	>–3
98	≥30 to <40	Classic	P40L	>–3
99	≥30 to <40	Classic	insertion	>–3
100	≥40 to <50	Classic	G360C	>–3
101	≥20 to <30	Non-Classic	P205S	>–3
102	≥40 to <50	No definite	G261D	>–3
103	≥50 to <60	No definite	L372R	>–3

eGFR, estimated glomerular filtration rate; ERT, enzyme replacement therapy; GVUS, genetic variants of unknown significance; ICF, informed consent form.
